# Renal Disease Burden in Prurigo Nodularis: A Systematic Review and Meta‐Analysis

**DOI:** 10.1111/ijd.70219

**Published:** 2025-12-22

**Authors:** Natalia Chalupczak, Natalie Bourand, Deborah Woo, Raj Chovatiya

**Affiliations:** ^1^ Chicago Medical School Rosalind Franklin University of Medicine and Science North Chicago Illinois USA; ^2^ College of Medicine, University of Illinois Chicago Chicago Illinois USA; ^3^ Center for Medical Dermatology + Immunology Research Chicago Illinois USA

**Keywords:** chronic kidney disease, end‐stage renal disease, inflammation, prurigo nodularis, pruritus, renal disease

**Abbreviations**
CI: confidence interval
CKD: chronic kidney disease
ESRD: end‐stage renal disease
ICD: international classification of diseases
PN: prurigo nodularis
RD: renal disease

1

Prurigo nodularis (PN) is a chronic pruritic dermatosis associated with neuroinflammatory dysregulation. Prior studies have suggested an increased comorbid burden of multiple conditions among PN patients, including cardiovascular and metabolic diseases. However, despite these more clearly described relationships [1], the relationship between PN and renal disease (RD) has not been systematically assessed. We conducted a systematic review and meta‐analysis to evaluate RD prevalence among patients with PN and summarize available evidence on PN occurring in RD populations.

Following Preferred Reporting Items for Systematic Reviews and Meta‐analyses (PRISMA) guidelines, multiple databases (PubMed, Embase, Scopus, Cochrane Library, and the Cumulative Index to Nursing and Allied Health Literature) were searched from inception to 08/08/2025 (https://data.mendeley.com/datasets/6s9xgw9ykk/1). Eligible studies included observational cohorts, cross‐sectional analyses, or case series of ≥ 10 patients reporting RD in PN populations or PN in RD cohorts available in English. Two independent reviewers (N.C. and N.B.) conducted title and abstract screening, full‐text review, data extraction, and quality assessment, with consensus reached in cases of disagreement.

Of 824 abstracts screened, 20 full texts were reviewed, and 17 studies were included in the final analysis (Figure 1A). Collectively, these studies represented over 143,179 patients with PN across North America, Europe, and Asia (Table 1). Multiple large databases and retrospective cohorts demonstrated an elevated prevalence of RD in PN patients. A multicenter United States cohort study of 12,157 PN patients reported chronic kidney disease (CKD) prevalence of 25.2% (*n* = 3063) and end‐stage renal disease (ESRD) in 6.0% (*n* = 729), while another United States cohort study of 7765 PN patients found ESRD in 16.3% (*n* = 1265). In a United Kingdom retrospective cohort study of 4,818 PN patients, 17.6% (*n* = 424) of 2,409 PN patients had CKD, with increased blood urea nitrogen (BUN) in 34.9% and Grade 2–4 eGFR abnormalities in 33.2%. Smaller cohorts from Spain (6.2%), China (6.6%), and the United States (8%–15%) also demonstrated heightened renal burden among PN patients.

**FIGURE 1:**
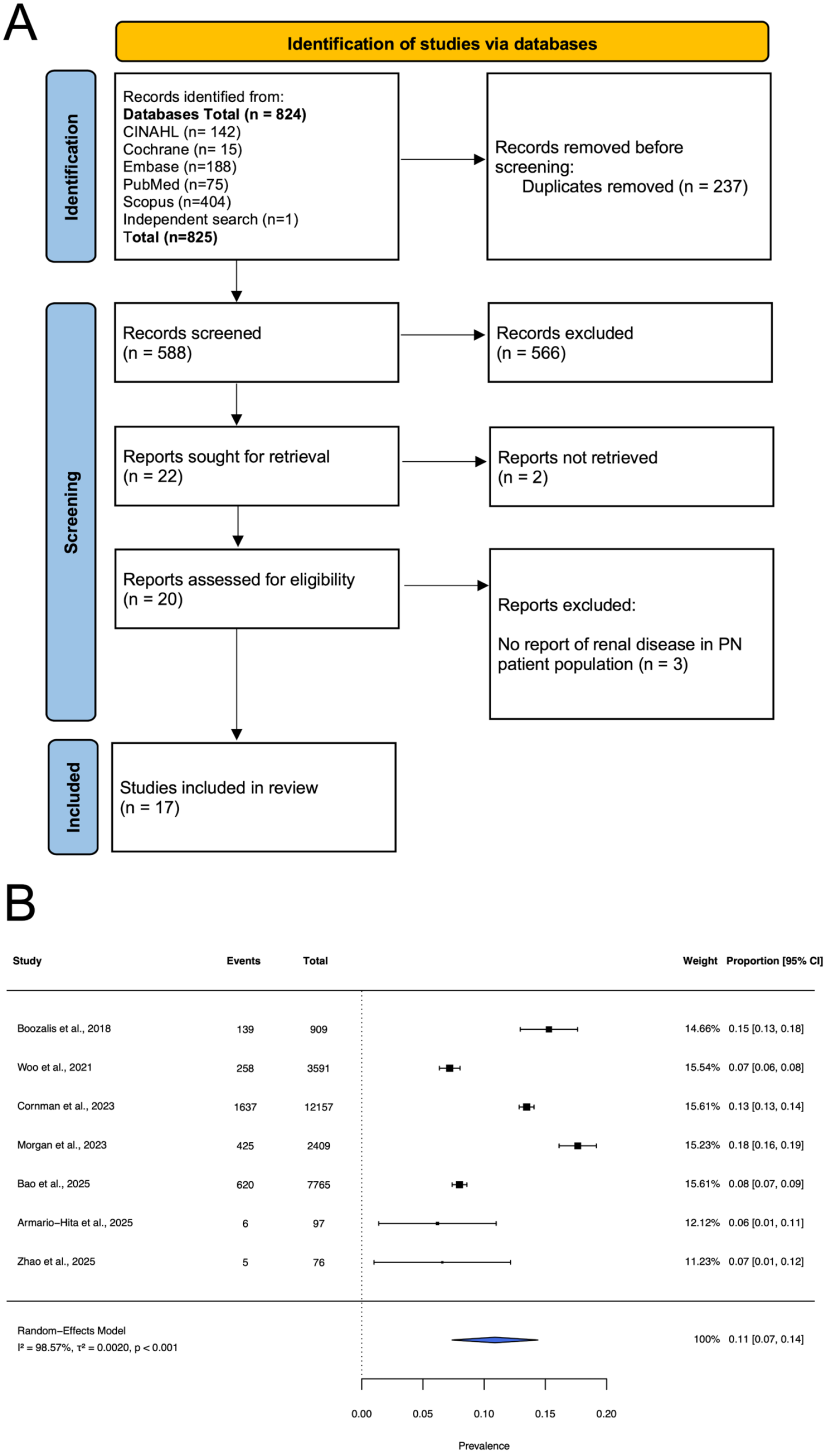
(A) Preferred Reporting Items for Systematic Reviews and Meta‐Analyses (PRISMA) flow diagram; (B) Random effects meta‐analysis of the prevalence of chronic kidney disease (CKD) in individuals with prurigo nodularis (PN).

**TABLE 1:** Characteristics of included studies examining prurigo nodularis and renal disease.

Patients with prurigo nodularis (PN)											
Study	Location	Design	Mean age of participants (years)	Total sample size	Patients with PN	Female patients, *n* (%)	PN patients with CKD, *n* (%)	PN patients on dialysis, *n* (%)	PN patients with ESRD, *n* (%)	PN patients with AKI, *n* (%)	Notes
Boozalis et al. 2018 [2]	United States (John Hopkins Hospital system)	Retrospective chart review	58 years (range 51–65)	909	909	450 (49.5)	139 (15.3)	NR	NR	NR	
Woo et al. 2021 [3]	Korea	Retrospective cross‐sectional	58.1 ± 19.4	17,955	3591	1551 (43.2)	258 (7.2)	NR	NR	NR	
Cornman et al. 2023 [4]	United States (TriNetX database)	Cross‐sectional	58.8 ± 15.4	24,314	12,157	7180 (59.1)	1637 (13.5)	NR	139 (6.0%)	NR	
Morgan et al. 2023 [5]	United Kingdom	Retrospective cohort	61.0 ± 18.4	4818	2409	1431 (59.4)	425 (17.6)	NR	NR	NR	
Williams et al. 2023 [6]	United States	Retrospective chart review	NR	57	57	NR	NR	NR	NR	NR	‐CKD reported as one of the 11 recurrent pruritic comorbid conditions in PN patients. ‐Exact prevalence of CKD not provided (*p* < 0.05)
Lee et al. 2022 [7]	United States (TriNetX database)	Cross‐sectional	NR	~69,000,000	NR	NR	NR	NR	NR	NR	‐PN strongly linked to CKD stages 1–5, ESRD, nephrotic syndrome, and multiple renal pathologies ‐CKD progression faster in Black PN patients (HR 2.88) with lower 10‐year survival rate (63.5% vs. 85.5% in Whites)
Czarnowicki et al. 2024 [8]	Israel	Cross‐sectional	NR	4197	4197	NR	NR	NR	NR	NR	‐Odd ratios of chronic hepatitis (OR 2.20) and dialysis (OR 2.11) in PN
Patel et al. 2024 [9]	United States (TriNetX database)	Retrospective cohort	55.2	92,965	92,965	54,013 (58.1)	NR	NR	NR	NR	‐PN was significantly associated with renal sclerosis (HR 5.0 [3.70–6.88]) compared to matched controls without PN
Elberling et al. 2025 [10]	Denmark	Nationwide retrospective cohort	59 ± 17	1209	1209	696 (58.0)	NR	NR	NR	NR	‐Before PN diagnosis, the highest OR was seen in CKD, with the PN group having an OR of 6.78 (95% CI 4.56–10.07) compared with the control group without PN
Mollanazar et al. 2025 [11]	United States	Retrospective, observational, descriptive cohort	55–56	17,729	17,729	NR	NR	NR	NR	NR	‐Reported as “renal disease” only: 9.7% of PN patients
Bao et al. 2025 [12]	United States	Cross‐sectional	56.1 ± 15.4	7765	7765	4085 (52.6)	620 (8.0)	NR	1265 (16.3)	1475 (19.0)	
Armario‐Hita et al. 2025 [13]	Spain	Cross‐sectional	53 ± 15.6	97	97	63 (65.0)	6 (6.2)	NR	NR	NR	
Zhao et al. 2025 [14]	China	Retrospective	50.9 ± 29.7	76	76	40 (52.6)	5 (6.6)	1 (1.3)	1 (1.3)	NR	

Patients with renal disease										
Study	Location	Design	Mean age of participants (years)	Total sample size	Patients with renal disease	Female patients, *n* (%)	CKD patients with PN, *n* (%)	Dialysis patients with PN, *n* (%)	ESRD patients with PN, *n* (%)	Notes
Hayani et al. 2016 [15]	Germany	Prospective cross‐sectional observational	64.7 ± 13.7	177	177	70 (39.5)	NR	18 (10.2)	18 (10.2)	‐Chronic itch cohort
Phan et al. 2009 [16]	Germany	Case series	66.7	18	18	4 (22.2)	NR	NR	NR	‐Uremic pruritis cohort: 13/18 were on dialysis overall; remaining had IgA nephritis, polycystic/sclerotic kidney, or diabetic nephritis
Kim et al. 2022 [17]	South Korea	Nationwide retrospective cohort	48.3 ± 14.3	17,295,576	897,481	8,385,033 (48.5)	NR	NR	NR	‐The study reports risk: compared with eGFR ≥ 90: eGFR 15–29 (HR 1.31 [1.05–1.62]) and ESRD (HR 1.46 [1.25–1.69]) ‐Proteinuria independently raised PN risk and amplified eGFR‐related risk: incidence of 3.60 per 10,000 person‐years ‐Renal disease n calculated by eGFR stratification.
Fathaddin, 2024 [18]	Saudi Arabia	Retrospective cross‐sectional	53.4 ± 19.8	44	44	25 (56.8)	5 (11.4)	NR	NR	−26/44 patients with CKD were from diabetic nephropathy (59.1%) −17 patients (38.6%) were receiving hemodialysis

Five studies directly evaluated PN among RD populations (Table 1). In a German cohort of 177 patients with chronic itch, 10.2% (*n* = 18) of patients with advanced CKD had clinically confirmed PN lesions. A smaller series of 18 uremic pruritus patients found PN in 66.7% of cases (*n* = 12).

Only reports examining CKD prevalence among patients with PN could be pooled for meta‐analysis given insufficient study and patient count for other comorbidities. Meta‐analysis was performed using the Metafor package in R. A standard random effects model (restricted maximum‐likelihood) was applied to estimate pooled prevalence with 95% confidence intervals (CIs). Among the seven included studies (total PN patients *n* = 27,004), the pooled prevalence of CKD among PN patients was 10.9% (95% CI: 7.4%–14.4%; *p* < 0.001) (Figure 1B). Heterogeneity across studies was high (*I* ^2^ = 98.57%, Q = 323.507, *p* < 0.001).

PN patients exhibited a relatively consistent burden of RD, especially CKD, which showed a pooled prevalence of 10.9% across seven studies, a figure that is slightly higher than a recent global CKD prevalence (9.5%) [19]. However, this similarity may not be interpretable as equivalence, as CKD severity may be a more relevant driver of heightened prevalence and/or association. Most included studies did not report CKD stage, age structure, or race/ethnicity, likely diluting risk‐enriched subgroups. Where staging was available, risk of GFR indeed increased with severity (eGFR 15–29: HR 1.31; ESRD: HR 1.46) (Table 1). In RD cohorts, PN prevalence ranged from 10.2% to 11.4%, severalfold higher than the ~0.07% baseline prevalence in the general population [20], further suggesting enrichment in this population as well.

Several limitations should be considered. The substantial heterogeneity likely reflects differences in RD definitions across studies, including laboratory‐defined CKD, ICD‐coded RD, or composite outcomes. Most studies were retrospective and relied on diagnostic codes, introducing a risk of misclassification for both PN and CKD. Important clinical data such as CKD stage, race/ethnicity, and age distribution were inconsistently reported, restricting the ability to perform stratified analyses. Publication bias could not be reliably assessed given the small number of eligible studies.

Mechanistically, several pathways may link PN with renal dysfunction. Uremic toxin accumulation in CKD promotes neuronal sensitization through IL‐31, TRPV1, and TRPA1 signaling, which may predispose individuals to chronic pruritus and nodule formation [21]. PN‐associated inflammation and tissue remodeling may contribute to microvascular and fibrotic dysregulation that exacerbates renal metabolic stress [22]. Shared metabolic comorbidities such as diabetes and hypertension likely further reinforce this overlap [1]. Future prospective studies stratified by CKD stage and other markers of disease activity are needed, as well as enhanced clinical surveillance and screening in these populations.

## Funding

The authors have nothing to report.

## Ethics Statement

This study did not requre ethics approval as it did not involved human participants, animal subjects, or identifiable personal data.

## Conflicts of Interest

R.C. has served as an advisor, consultant, speaker, and/or investigator for AbbVie, Acelyrin, Alumis, Amgen, AnaptysBio, Apogee Therapeutics, Arcutis Biotherapeutics Inc., Argenx, Astria Therapeutics Inc., Avalere Health, Beiersdorf, Boehringer Ingelheim, Bristol Myers Squibb, Cara Therapeutics, Castle Biosciences, CLn Skin Care, Dermavant, Eli Lilly and Company, EMD Serono, Formation Bio, Galderma, Genentech, GSK, Incyte, Johnson & Johnson, Kenvue, LEO Pharma, L'Oréal, Nektar Therapeutics, Novartis, Opsidio, Pfizer Inc., RAPT, Regeneron, Sanofi, Sitryx, Takeda, TRex Bio, UCB, Zai Lab. N.C., N.B., and D.W. have no conflicts of interest to declare.

## Data Availability

All data supporting the findings of this study are available within the manuscript. Additional datasets extracted from published articles and used in this meta‐analysis are available from the corresponding author upon reasonable request.
